# Association between cotinine-verified smoking status and moderately increased albuminuria in the middle-aged and older population in Korea: A nationwide population-based study

**DOI:** 10.1371/journal.pone.0246017

**Published:** 2021-02-10

**Authors:** Yeonjoo Choi, Joo-Hyun Park, Do-Hoon Kim, Hyun Jin Kim, Euijung Suh, Ki-Hoon Kim, Jae Joon Ahn, Gyu-Na Lee, Jin-Hyung Jung, Kyungdo Han, You-Na Shin

**Affiliations:** 1 Department of Family Medicine, Korea University Ansan Hospital, Korea University College of Medicine, Ansan, Republic of Korea; 2 Department of Biostatistics, College of Medicine, The Catholic University of Korea, Seoul, Republic of Korea; 3 Department of Statistics and Actuarial Science, Soongsil University, Seoul, Republic of Korea; 4 Korea Medical Institute Suwon Center, Suwon, Republic of Korea; University of Mississippi Medical Center, UNITED STATES

## Abstract

**Objectives:**

Although several self-reported questionnaire-based studies have found an association between smoking and moderately increased albuminuria, this result remains controversial. We investigated whether moderately increased albuminuria was associated with smoking status, verified by urinary cotinine (an objective biomarker of tobacco exposure), using population-based, nationally representative data.

**Methods:**

This study included 2059 participants aged ≥ 50 years from the 2014 Korean National Health and Nutrition Examination Survey. Individuals with a urinary cotinine level ≥ 50 ng/mL were identified as cotinine-verified smokers. Moderately increased albuminuria was defined as a urine albumin-to-creatinine ratio ranging between ≥ 30 mg/g and < 300 mg/g. Multivariable logistic regression was used to evaluate the association between cotinine-verified smoking status and moderately increased albuminuria.

**Results:**

Among the study participants, 16.9% were cotinine-verified smokers, 84.8% of whom were men. After adjustment for multiple covariates, cotinine-verified smokers showed a significant positive association with moderately increased albuminuria (adjusted odds ratio: 4.37, 95% confidence interval: 1.63–11.71) compared with cotinine-verified non-smokers. The association between urinary cotinine and moderately increased albuminuria did not differ with age, sex, obesity, or comorbidities (P-value for interaction > 0.05 in all cases).

**Conclusion:**

This large-scale observational study showed that cotinine-verified smoking is associated with moderately increased albuminuria in the Korean middle-aged and older general population, suggesting that smoking must be strictly controlled to reduce the risk of moderately increased albuminuria.

## Introduction

As the aging population grows rapidly, the prevalence of chronic diseases such as cardiovascular disease and chronic kidney disease is increasing concomitantly [1, 2]. The social and financial burdens of these diseases are expected to rise [3, 4]. Therefore, researchers, clinicians, and policymakers must strive extensively to prepare for and lower this burden.

In this regard, moderately increased albuminuria is a powerful predictor of cardiovascular and chronic kidney disease, especially in middle-aged and older populations. Albuminuria is a well-known cardiovascular risk factor [5, 6] and is the earliest marker of glomerular disease [7]. Current guidelines suggest that patients with moderately increased albuminuria are at an increased risk for chronic kidney disease progression [7]. Conversely, studies have shown that reduction in albuminuria has renal [8] as well as cardiovascular protective effects [9]. In a Dutch general population study, screening and treatment of albuminuria were found to be more cost-effective than no screening [10]. It follows that early detection and preemptive intervention in subjects with moderately increased albuminuria may prevent the progression of chronic kidney disease and cardiovascular disease.

There is growing evidence that smoking is a preventable risk factor for the decline in kidney function [11–14]. However, results regarding the association between smoking and moderately increased albuminuria are inconsistent, with some studies presenting a positive association [15–20] and others showing no association [21]. Notably, most studies have monitored smoking status using self-reported questionnaires or interviews [15–19, 21], which could lead to underestimation because subjects tend to deny tobacco use [22]. Furthermore, most studies have found that smoking is associated with albuminuria only in specific sectors of the population, such as patients with diabetes [15–17, 20] and men [18, 19].

The main methods used to assess smoking status are questionnaires and cotinine measurements. Cotinine is a nicotine metabolite that can be used to quantify tobacco exposure [23]. It is a more reliable indicator of tobacco exposure than self-reporting [24]. Only a few Western studies have used urinary cotinine levels to verify smoking status [20, 25, 26], and no Asian study has investigated the association between cotinine-verified smoking status and moderately increased albuminuria.

Therefore, in this study we investigated the association between urinary cotinine-verified smoking status (an objective biomarker of tobacco exposure) and moderately increased albuminuria in the general middle-aged and older populations. We used population-based nationally representative data collected from the Korea National Health and Nutrition Examination Survey (KNHANES).

## Materials and methods

### Study population

We collected data from the KNHANES VI: a cross-sectional survey conducted by the Korea Centers for Disease Control and Prevention (KCDC) since 1998. The data from this survey include detailed information on individuals’ medical history, socio-demographic attributes, and nutritional status. In this study, we used data from the 2014 KNHANES, and the study population comprised adults aged ≥ 50 years.

**Fig 1:**
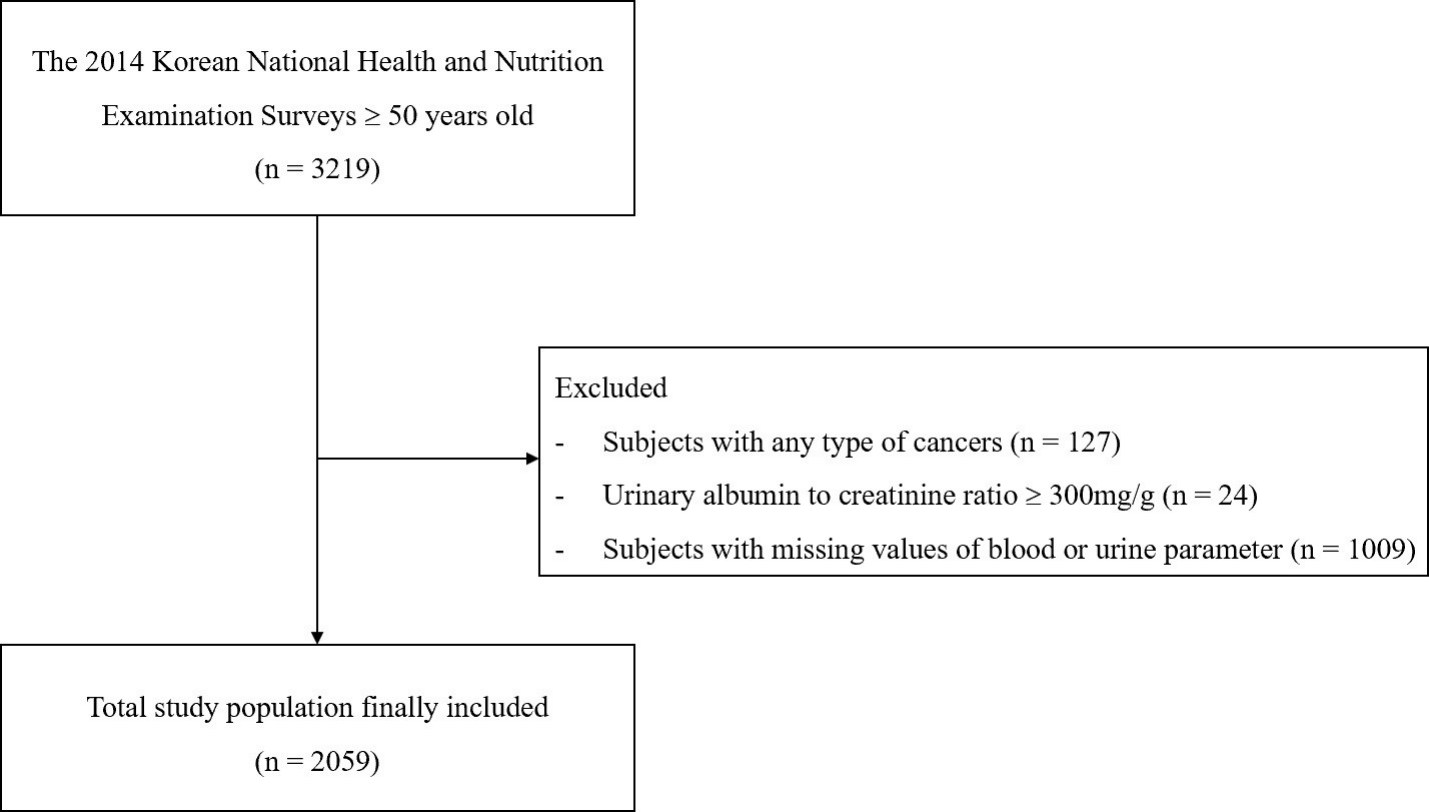
Flow diagram outlining the inclusion and exclusion criteria and the study design.

Among the 7550 participants included in the 2014 KNHANES, 3219 were aged 50 years or older. We excluded 1009 subjects with missing data on blood and urine parameters, including urine cotinine level, moderately increased albuminuria, and urine creatinine level. Forty-nine subjects with a urinary albumin-to-creatinine ratio (UACR) ≥ 300 mg/g and 127 patients with any type of cancer were excluded because of the possibility of exhibiting moderately increased albuminuria. Ultimately, 2059 subjects were analyzed in the present study (Fig 1). All personally identifiable information has been removed from the data to allow for an anonymous analysis and written informed consent was obtained from all participants in KNHANES. The study protocol was approved by the institutional review board of KCDC (2013-12EXP-03-5C).

### Measurement of urinary cotinine

Spot urine samples were collected from the subjects in the morning from a midstream void. Urinary cotinine levels were measured by gas chromatography–mass spectrometry with a Perkin Elmer Clarus 600T gas chromatograph/mass spectrometer (PerkinElmer, Turku, Finland). We defined participants with urinary cotinine levels below 50 ng/mL as cotinine-verified non-smokers and those with urinary cotinine levels ≥ 50 ng/mL as cotinine-verified smokers, as stated in previous studies [27–29].

### Measurement and definition of moderately increased albuminuria

For the assessment of moderately increased albuminuria, the same samples of spot urine collected for urinary cotinine measurement were used. Urinary albumin levels were measured by the turbidimetric assay using the Hitachi Automatic Analyzer 7600 (Hitachi, Tokyo, Japan). Urinary creatinine levels were measured by the Jaffe rate-blanked and compensated method using the Hitachi Automatic Analyzer 7600–210 (Hitachi, Tokyo, Japan). UACR was expressed as milligrams of urinary albumin per gram of urinary creatinine (mg/g). Moderately increased albuminuria was defined as a UACR ≥ 30 mg/g and < 300 mg/g [30, 31].

### Demographic and lifestyle variables

Information on age, sex, and comorbidities was obtained using a health questionnaire. Body mass index (BMI) was calculated as body weight in kg divided by height in m², both of which were measured by trained medical technicians following standardized protocols. Participants with comorbidities such as hypertension, diabetes mellitus, dyslipidemia, and liver cirrhosis were defined as those who answered that they were currently diagnosed as having the respective diseases.

Educational level was identified as the percentage of high school graduates, and monthly household income was defined as the percentage of those with the lowest quartile income level. Alcohol consumption and smoking were presented as the percentage of participants who consumed alcohol more than once a month in a year and those who answered that they were current smokers, respectively. Regular physical activity was defined as ≥ 2.5 hours per week of moderate-intensity physical activity (moderate shortness of breath or moderate acceleration of heart rate), ≥ 1 h and 15 min of vigorous-intensity physical activity per week (rapid breathing and substantial acceleration of heart rate) or mixed-intensity physical activity for the equivalent amount of time, with 1 min of vigorous-intensity physical activity being equal to 2 min of moderate-intensity physical activity.

### Anthropometry and biological measurements

Anthropometric measurements were performed by trained personnel. Body height and body weight were measured using standardized procedures to the nearest 0.1 cm and 0.1 kg, respectively, with the subjects dressed in light clothing without shoes. Obesity was defined as a BMI ≥ 25 kg/m^2^ according to Asian standards. Blood pressure was measured using a mercury sphygmomanometer, with the participants in the sitting position and having rested for 5 min. After three measurements, the average of the latter two measurements was used as the final data. Blood samples were obtained after ≥ 8 h of fasting; they were transported in cold storage to the Central Testing Institute (Seoul, South Korea) and analyzed within 24 h. The serum level of total cholesterol was measured using a Hitachi Automatic Analyzer 7600–210 (Hitachi, Tokyo, Japan), with commercially available kits (Sekisui, Japan).

### Statistical analysis

The baseline characteristics were compared between cotinine-verified smokers and non-smokers and presented as mean ± standard deviation for continuous variables or number with percentages for categorical data. P-values were calculated using Student’s t-test for continuous variables and the chi-squared test for categorical variables. Multivariable logistic regression analysis was carried out to evaluate the relationship between moderately increased albuminuria and urine cotinine levels. Odds ratios (ORs) and 95% confidence intervals (CIs) were assessed after adjustment for multiple covariates. In model 1, age and sex were the adjusted covariates. We additionally adjusted for BMI, income level, education, alcohol consumption, smoking status, and physical activity in model 2. In model 3, we additionally adjusted for comorbidities. We also conducted a subgroup analysis of cotinine-verified smokers and moderately increased albuminuria according to age, sex, BMI, diabetes mellitus, hypertension, dyslipidemia, and chronic kidney disease.

All statistical analyses were performed using SAS version 9.3 software (SAS Institute, Cary, NC, USA). Two-sided P-values < 0.05 were considered statistically significant.

## Results

### Baseline characteristics

The baseline clinical characteristics of the study participants are shown in Table 1. Of the 2059 participants included, 349 (16.9%) were cotinine-verified smokers and 1710 (83.1%) were cotinine-verified non-smokers. Compared with the cotinine-verified non-smokers, the cotinine-verified smokers were younger (P < 0.001), included a higher proportion of male participants (84.8%, P < 0.001), were more likely to consume alcohol (P < 0.001), and had a lower mean BMI (P < 0.001). They also had higher levels of urine and serum creatinine (P < 0.001), aspartate aminotransferase (P = 0.008), and alanine aminotransferase (P = 0.008). More individuals were diagnosed as having diabetes mellitus (P = 0.014), and fewer individuals were diagnosed as having hypertension (P = 0.025) and hyperlipidemia (P = 0.003) in the cotinine-verified smoker group.

**Table 1:** Baseline characteristics of the participants according to urinary cotinine level.

	Total		Cotinine-verified non-smoker		Cotinine-verified smoker		*P*-value
	(n = 2059)		(n = 1710)		(n = 349)		
Age, years	63.2	±8.3	63.5	±8.4	61.4	±7.7	< .001
Sex, male, %	889	(43.2)	593	(34.7)	296	(84.8)	< .001
Body mass index, mean, kg/m^2^	24.0	±3.1	24.2	±3.0	23.4	±3.1	< .001
SBP, mmHg	122.7	± 16.7	123.0	±16.9	121.3	±15.5	0.073
DBP, mmHg	75.3	± 10.1	75.3	±10.0	75.2	±10.8	0.830
Urine creatinine, mg/dl	125.1	± 69.1	120.0	±65.5	150.0	±80.3	< .001
Urine microalbumin, μg/dl	1831.3	±3774.3	1796.9	±3790.5	2000.3	±3694.5	0.359
BUN, mg/dl	15.8	±4.2	15.9	±4.3	15.5	±4.2	0.135
Creatinine, mg/dl	0.83	±0.2	0.81	±0.19	0.92	±0.2	< .001
ALT, IU/L*	19.0	(18.7,19.4)	18.8	(18.4,19.2)	20.2	(19.2,21.3)	0.008
AST, IU/L*	22.5	(22.2,22.9)	22.4	(22.0,22.7)	23.5	(22.5,24.5)	0.008
Fasting plasma glucose level, mg/dL	103.6	±23.1	103.1	±22.6	106.3	±25.0	0.016
Total cholesterol level, mg/dL	191.3	±35.7	191.9	±35.5	188.4	±36.7	0.092
Diabetes mellitus	366	(17.8)	288	(16.8)	78	(22.4)	0.014
Hypertension,	1121	(54.4)	950	(55.6)	171	(49)	0.025
Hyperlipidemia,	487	(23.7)	426	(24.9)	61	(17.5)	0.003
Pulmonary disease	48	(2.3)	41	(2.4)	7	(2.0)	0.658
Cardiovascular disease	71	(3.5)	59	(3.5)	12	(3.4)	0.991
Liver cirrhosis	6	(0.3)	4	(0.2)	2	(0.6)	0.284
Income (Lowest quartile)	550	(26.7)	461	(27.0)	89	(25.5)	0.575
Education (≥high school)	871	(42.3)	713	(41.7)	158	(45.3)	0.218
Alcohol consumption	938	(45.6)	697	(40.8)	241	(69.1)	< .001
Physical activity	997	(48.4)	832	(48.7)	165	(47.3)	0.639

Data are presented as mean ± standard deviation or number (percentage).

*Geometric mean (95% confidence interval)

Cotinine-verified smokers were defined as those with urine cotinine ≥50 ng/mL, and cotinine-verified non-smokers were defined as those with urine cotinine <50 ng/mL.

ALT, alanine aminotransferase; AST, aspartate aminotransferase; BUN, blood urea nitrogen; DBP, diastolic blood pressure; SBP, systolic blood pressure

### Risk of moderately increased albuminuria according to urinary cotinine level

Table 2 shows the ORs and 95% CIs of the multivariable adjusted logistic regression between urinary cotinine levels and moderately increased albuminuria. We also calculated the ORs and 95% CIs of the covariates.

**Table 2:** Multivariable adjusted odds ratios of moderately increased albuminuria according to cotinine-verified smoking status.

	Odds ratio (95% confidence interval)					
	Model 1		Model 2		Model 3	
Smoking status						
Cotinine-verified Non-smokers	1	[Reference]	1	[Reference]	1	[Reference]
Cotinine-verified Smokers	1.17	(0.71–1.94)	3.42	(1.32–8.89)	4.37	(1.63–11.71)
*P*-value		0.545		0.012		0.004
Age						
Per 1 year	1.04	(1.02–1.06)	1.04	(1.02–1.07)	1.03	(1.002–1.05)
*P*-value		<0.001		<0.001		0.032
Sex						
Women	1	[Reference]	1	[Reference]	1	[Reference]
Men	0.95	(0.65–1.39)	0.96	(0.64–1.45)	0.91	(0.59–1.41)
*P*-value		0.780		0.854		0.679
Body mass index						
Per 1 kg/m^2^			1.13	(1.06–1.20)	1.09	(1.02–1.16)
*P*-value				<0.001		0.009
Income level						
Second to forth quartile			1	[Reference]	1	[Reference]
Lowest quartile			1.08	(0.73–1.60)	1.03	(0.70–1.53)
*P*-value				0.712		0.866
Education						
No			1	[Reference]	1	[Reference]
Yes			1.03	(0.76–1.40)	1.08	(0.79–1.47)
*P*-value				0.839		0.643
Alcohol consumption						
No			1	[Reference]	1	[Reference]
Yes			1.06	(0.72–1.56)	0.98	(0.65–1.48)
*P*-value				0.761		0.937
Physical activity						
No			1	[Reference]	1	[Reference]
Yes			1.04	(0.72–1.50)	1.08	(0.75–1.58)
*P*-value				0.832		0.669
Hypertension						
No					1	[Reference]
Yes					2.27	(1.53–3.36)
*P*-value						<0.001
Diabetes						
No					1	[Reference]
Yes					2.61	(1.82–3.73)
*P*-value						<0.001
Hyperlipidemia						
No					1	[Reference]
Yes					0.97	(0.67–1.39)
*P*-value						0.845
Pulmonary disease						
No					1	[Reference]
Yes					0.49	(0.18–1.36)
*P*-value						0.170
Cardiovascular disease						
No					1	[Reference]
Yes					0.85	(0.41–1.76)
*P*-value						0.652
Liver cirrhosis						
No					1	[Reference]
Yes					1.41	(0.20–10.15)
*P*-value						0.731

Cotinine-verified smokers were defined as those with urine cotinine ≥50 ng/mL, and cotinine-verified non-smokers were defined as those with urine cotinine <50 ng/mL.

Model 1: adjusted for age, sex.

Model 2: adjusted for age, sex, body mass index, income level, education, alcohol consumption, smoking status, and physical activity.

Model 3: adjusted for age, sex, body mass index, income level, education, alcohol consumption, smoking status, physical activity, hypertension, diabetes mellitus, hyperlipidemia, pulmonary disease, cardiovascular disease, and liver cirrhosis.

There was no significant association between urinary cotinine level and moderately increased albuminuria on adjustment for age and sex. However, after additionally adjusting for BMI, income level, education, alcohol consumption, smoking status, and physical activity (model 2), cotinine-verified smokers had a higher risk of moderately increased albuminuria, with an OR of 3.42 (95% CI: 1.32–8.89), than did cotinine-verified non-smokers. After additionally adjusting for comorbidities, such as hypertension, diabetes, hyperlipidemia, pulmonary disease, cardiovascular disease, and liver cirrhosis, the OR of moderately increased albuminuria was 4.37 (95% CI: 1.63–11.71) in the group of cotinine-verified smokers. Among the covariates, age, obesity, hypertension, and diabetes showed a significant association with moderately increased albuminuria. Older age, obesity, hypertension, and diabetes showed an OR of 1.03 (95% CI: 1.002–1.05), 1.09 (95% CI: 1.02–1.16), 2.27 (95% CI: 1.53–3.36), and 2.61 (95% CI: 1.82–3.72), respectively, when adjusted for model 3.

When we conducted multivariable adjusted logistic regression between urinary cotinine and albuminuria, which we defined as UACR >30 mg/g, the result showed a similar tendency. Although there was no significant association between urinary cotinine level and albuminuria upon adjustment for age and sex, there was a significant association upon adjustment for models 2 and 3, with an OR of 3.36 (95% CI: 1.30–8.67) and 4.17 (95% CI: 1.57–11.05), respectively. The covariates also showed a similar association. This result is presented in S1 Table.

**Fig 2:**
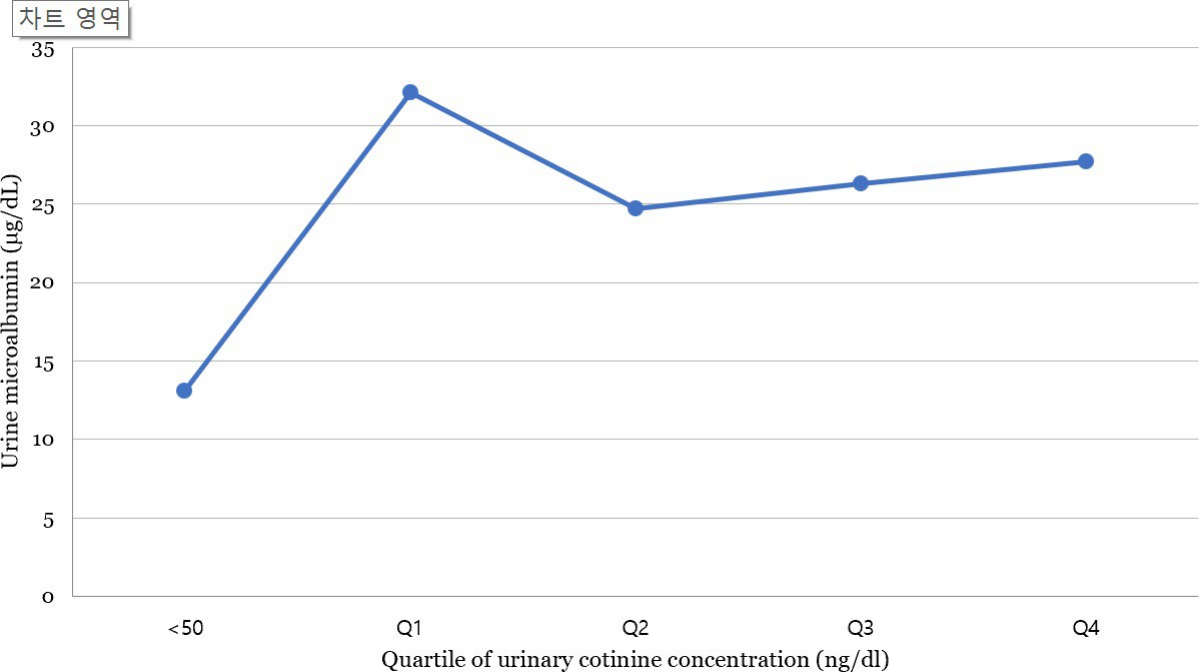
Urine microalbumin level according to urinary cotinine levels, adjusted for age, sex, body mass index, income level, education, alcohol consumption, smoking status, physical activity, hypertension, diabetes mellitus, hyperlipidemia, pulmonary disease, cardiovascular disease, and liver cirrhosis.

Fig 2 shows the mean urine microalbumin level of the participants according to urinary cotinine level. The mean urine microalbumin level was 13.09 μg/dl in the cotinine-verified non-smoker group, whereas in the first (Q1), second (Q2), third (Q3), and fourth quartiles (Q4) of the cotinine-verified smoker group, the levels were 32.1, 24.7, 26.3, and 27.7 μg/dl, respectively when the subjects were stratified according to urinary cotinine level. The urine microalbumin level was higher in cotinine-verified smokers than in cotinine-verified non-smokers. As the urine cotinine level increased from Q1 to Q4, the urine microalbumin level did not show an increasing trend (P for trend = 0.35).

### Subgroup analysis

Table 3 shows the results of the subgroup analysis of urinary cotinine levels and moderately increased albuminuria according to age, sex, BMI, diabetes mellitus, hypertension, dyslipidemia, and chronic kidney disease. The association between urine cotinine level and moderately increased albuminuria did not vary according to any of these factors (P-values for interaction: age, 0.866; sex, 0.560; BMI, 0.155; diabetes mellitus, 0.456; hypertension, 0.486; dyslipidemia, 0.721; and chronic kidney disease, 0.121).

**Table 3:** Association between cotinine-verified smokers and moderately increased albuminuria according to subgroup.

		Adjusted* odds ratio (95% confidence interval)		*P*-value for interaction
Age, years	50–64	6.51	(2.09–20.3)	0.866
	≥65	1.75	(0.32–9.59)	
Sex	Male	6.2	(1.55–24.8)	0.560
	Female	2.92	(0.65–13.1)	
Obesity	Yes	3.39	(0.65–17.6)	0.155
	No	4.34	(1.34–14.0)	
Diabetes	Yes	5.47	(1.46–20.6)	0.456
	No	4.44	(1.29–15.3)	
Hypertension	Yes	5.14	(1.83–14.5)	0.486
	No	1.16	(0.29–4.63)	
Dyslipidemia	Yes	4.54	(1.57–13.2)	0.721
	No	2.58	(0.46–14.5)	
Chronic kidney disease	Yes	4.59	(1.67–12.6)	0.121
	No	0.90	(0.004–191.8)	

*Adjusted for age, sex, body mass index, income level, education, alcohol consumption, smoking status, physical activity, hypertension, diabetes mellitus, hyperlipidemia, pulmonary disease, cardiovascular disease, and liver cirrhosis.

Obesity was defined as body mass index ≥ 25 kg/m^2^.

## Discussion

In this nationwide study of the Korean general population over 50 years old, we demonstrated a 4-fold higher association of moderately increased albuminuria among smokers compared to non-smokers, as verified by urinary cotinine levels. The association between urinary cotinine level and moderately increased albuminuria was consistent regardless of age, sex, BMI, and other comorbidities. This is the first Asian study to evaluate the association between cotinine-verified smoking and moderately increased albuminuria in the general middle-aged and older population using urine cotinine as an objective and quantitative measure of smoking.

Our results are consistent with those of prior studies in terms of the association between smoking and moderately increased albuminuria. Previous studies have found that the OR or hazard ratio for moderately increased albuminuria in smokers compared to that in non-smokers is between 1.4 and 4.5 in Asian countries [18, 32, 33] and between 1.3 and 4.9 in Western countries [16, 17, 34–40]. However, the study population only comprised patients with diabetes [16–18, 32, 34, 36], patients at a high risk of cardiovascular disease [38], or men [39]. Moreover, all of the abovementioned studies analyzed smoking status as reported in a questionnaire or interview. In contrast, a study conducted among the general Korean population showed no association between smoking and moderately increased albuminuria, as measured by the dip-stick method, which is prone to inaccuracies [21]. Only a few studies have used serum cotinine levels to identify participants’ smoking status, and these either had a small study population [26] or showed a positive association between smoking and moderately increased albuminuria only in participants with hypertension [25].

Several hypotheses have been proposed to explain the mechanism underlying the association between smoking and moderately increased albuminuria. Some studies have suggested that nicotine increases sympathetic activity by stimulating catecholamine release from peripheral sympathetic nerve endings [41, 42]. Alternatively, vasodilatation may be impaired due to endothelial cell damage in smokers [43, 44]. Endothelial cell dysfunction has been implicated in the pathogenesis of glomerular injury and consequent altered renal hemodynamics. Moreover, smokers tend to be more thrombogenic with enhanced platelet activity and thromboxane metabolism. Other potential mechanisms of smoking-induced kidney damage include oxidative stress, antidiuretic action of nicotine, and increased renal vascular tone due to decreased nitric oxide bioavailability in smokers [42]. These factors may have a cumulative effect on the overall condition of the kidney. Smoking may also affect hypertension, diabetes, and hyperlipidemia, which are risk factors for moderately increased albuminuria. Smoking also causes interruption of glucose homeostasis, increasing the risk of type 2 diabetes mellitus [45]. Also, smoking is associated with an increased risk of dyslipidemia and also causes transient elevation of blood pressure [27, 46, 47]. However, we found a significant association between cotinine-verified smoking and moderately increased albuminuria even after adjusting for various covariates, including hypertension, diabetes, and hyperlipidemia. In addition, the interactions with diabetes, hypertension, and hyperlipidemia were not significant in the subgroup analysis. In subjects without diabetes or hypertension, there was a significant association between cotinine-verified smoking and moderately increased albuminuria. Therefore, we can suggest that smoking has an independent association with moderately increased albuminuria.

In our study, the urinary cotinine level and urine microalbumin level did not show a dose–response relationship. An increase in urine cotinine levels indicates a high intensity of current smoking. Although the smoking status determined by the urine cotinine level is more accurate than that determined using the questionnaire, it does not reflect the cumulative effect for a period [48]. Therefore, there may not be a dose–response relationship between urinary cotinine levels and urine microalbumin levels. However, even low urinary cotinine levels were significantly associated with increased urine microalbumin levels, suggesting that even low-intensity smoking is significantly associated with albuminuria.

The strength of the present study lies in the use of a nationally representative database to demonstrate an association between smoking and moderately increased albuminuria in the Korean population. Moreover, by utilizing urinary cotinine as a biochemical marker, we quantified smoking status and eliminated underestimation. Finally, we adjusted the data for multiple confounding variables, including income level, education level, alcohol consumption, physical activities, and major comorbidities.

The limitations of the present study are as follows. First, we did not consider the possible impact of nicotine replacement therapy on urinary concentrations of cotinine. Second, we conducted this study in middle-aged and older people because the prevalence of moderately increased albuminuria in young people is relatively very low. As our findings are limited to middle-aged and older people, we did not conclude the association between cotinine-verified smoking and moderately increased albuminuria in young people. Third, as this was a cross-sectional study, the longitudinal association between urinary cotinine and moderately increased albuminuria was not evaluated. Fourth, we attempted to mitigate confounding bias by adjusting for a wide range of covariates. Nevertheless, it is possible that some residual confounding remains.

## Conclusions

In conclusion, we found a significant association between cotinine-verified smoking and moderately increased albuminuria in the South Korean middle-aged and older general population. Our findings suggest the need for strict control of smoking to reduce the risk of moderately increased albuminuria. Additional longitudinal studies are needed to elucidate the impact of smoking and smoking cessation on the development of moderately increased albuminuria.

## Supporting information

S1 Table Multivariable adjusted odds ratios of albuminuria according to cotinine-verified smoking status. (PDF)

S1 Dataset (DOCX)
