# Supplementary material for: Association between cotinine-verified smoking status and moderately increased albuminuria in the middle-aged and older population in Korea: A nationwide population-based study
Source: PLoS One. 2021 Feb 10;16(2):e0246017. doi: 10.1371/journal.pone.0246017 (PMC7875375; doi:10.1371/journal.pone.0246017)
Supplement: S1 Table — (PDF) [file pone.0246017.s001.pdf]

	Odds ratio (95% confidence interval)					
	Model 1		Model 2		Model 3	
Smoking status						
Cotinine-verified non-smokers		[Reference]	1	[Reference]	1	[Reference]
Cotinine-verified smokers	1.15	(0.70-1.90)	3.36	(1.30-8.67)	4.17	(1.57-11.05)
P-value	0.584		0.013		0.004	
Age						
per 1 year	1.04	(1.02-1.06)	1.04	(1.02-1.07)	1.03	(1.003-1.05)
P-value	<0.001		<0.001		0.029	
Sex						
Women	1	[Reference]	1	[Reference]	1	[Reference]
Men	0.94	(0.64-1.37)	0.95	(0.64-1.43)	0.88	(0.57-1.35)
P-value	0.747		0.813		0.555	
Body mass index						
per 1 kg/m^2			1.13	(1.06-1.19)	1.09	(1.02-1.16)
P-value			<0.001		0.011	
Income level						
Second to forth quartile			1	[Reference]	1	[Reference]
Lowest quartile			1.06	(0.72-1.58)	1.02	(0.68-1.52)
P-value			0.763		0.919	
Education						
No			1	[Reference]	1	[Reference]
Yes			1.04	(0.77-1.40)	1.09	(0.80-1.49)
P-value			0.815		0.577	
Alcohol consumption						
No			1	[Reference]	1	[Reference]
Yes			1.07	(0.73-1.57)	0.98	(0.65-1.48)
P-value			0.735		0.930	
Physical activity						
No			1	[Reference]	1	[Reference]
Yes			1.04	(0.72-1.50)	1.08	(0.74-1.57)
P-value			0.837		0.692	
Hypertension						
No					1	[Reference]
Yes					2.22	(1.51-3.27)
P-value					<0.001	
Diabetes						
No					1	[Reference]
Yes					2.54	(1.77-3.64)
P-value					<0.001	
Hyperlipidemia						
No					1	[Reference]

Yes	0.94	(0.66-1.34)
P-value	0.722	
Pulmonary disease		
No	1	[Reference]
Yes	0.49	(0.18-1.37)
P-value	0.172	
Cardiovascular disease		
No	1	[Reference]
Yes	0.77	(0.36-1.66)
P-value	0.509	
Liver cirrhosis		
No	1	[Reference]
Yes	1.49	(0.21-10.84)
P-value	0.691	

Albuminuria was defined as urinary albumin-to-creatinine ratio ≥30 mg/g.

Cotinine-verified smokers were defined as urine cotinine ≥50 ng/mL, and cotinine-verified non-smokers were defined as urine cotinine <50 ng/mL.

Model 1: adjusted for age, sex.

Model 2: adjusted for age, sex, body mass index, income level, education, alcohol consumption, smoking status, and physical activity.

Model 3: adjusted for age, sex, body mass index, income level, education, alcohol consumption, smoking status, physical activity, hypertension, diabetes mellitus, hyperlipidemia, pulmonary disease, cardiovascular disease, and liver cirrhosis.
