# Supplementary material for: Association between cotinine-verified smoking status and moderately increased albuminuria in the middle-aged and older population in Korea: A nationwide population-based study
Source: PLoS One. 2021 Feb 10;16(2):e0246017. doi: 10.1371/journal.pone.0246017 (PMC7875375; doi:10.1371/journal.pone.0246017)
Supplement: S1 Dataset — (DOCX) [file pone.0246017.s002.docx]

**Minimal data set**

The values used to build the graph in Fig 2.

| Urinary cotinine level | The adjusted-mean* urine microalbumin level (ug/dl) |
| --- | --- |
| <50 ng/dl | 13.1 |
| 1^st^ Quartile | 32.1 |
| 2^nd^ Quartile | 24.7 |
| 3^rd^ Quartile | 26.3 |
| 4^th^ Quartile | 27.7 |
| *P* for trend | 0.349 |

*adjusted for age, sex, body mass index, income level, education, alcohol consumption, smoking status, physical activity, hypertension, diabetes mellitus, hyperlipidemia, pulmonary disease, cardiovascular disease, and liver cirrhosis.
